# Dimethyl 2,6,8-trimethyl-1,2-dihydroquinoline-2,4-dicarboxylate

**DOI:** 10.1107/S160053681004153X

**Published:** 2010-10-23

**Authors:** Zeynep Gültekin, Wolfgang Frey, Barış Tercan, Tuncer Hökelek

**Affiliations:** a Department of Chemistry, Çankırı Karatekin University, TR-18100, Çankırı, Turkey; b Universität Stuttgart, Pfaffenwaldring 55, D-70569, Stuttgart, Germany; c Department of Physics, Karabük University, 78050 Karabük, Turkey; d Department of Physics, Hacettepe University, 06800 Beytepe, Ankara, Turkey

## Abstract

The title compound, C_16_H_19_NO_4_, the hydrogenated ring adopts a twisted conformation. In the crystal, inter­molecular C—H⋯O hydrogen bonds link the mol­ecules into centrosymmetric *R*_2_^2^(10) dimers. These dimers are further connected *via* inter­molecular N—H⋯O hydrogen bonds, forming infinite double chains along [001].

## Related literature

For the preparation of 1,2-dihydro­quinoline, see: Edwards *et al.* (1998 ▶); Yan *et al.* (2004 ▶); Petasis & Butkevich (2009 ▶); Johnson *et al.* (1989 ▶); Waldmann *et al.* (2008 ▶); Rueping & Gültekin (2009 ▶). For the biological activity of dihydro­quinolines, see: Elmore *et al.* (2001 ▶); Dillard *et al.* (1973 ▶); Muren & Weissmann (1971 ▶). For the preparation of quinolines, see: Dauphinee & Forrest (1978 ▶); Yan *et al.* (2004 ▶); Tom & Ruel (2001 ▶); Tokuyama *et al.* (2001 ▶); Sarma & Prajapati (2008 ▶); Martinez *et al.* (2008 ▶); Huang *et al.* (2009 ▶); Katritzky *et al.* (1996 ▶). For the biological activity of quinolines, see: Hamann *et al.* (1998 ▶); He *et al.* (2003 ▶); LaMontagne *et al.* (1989 ▶). For graph-set analysis, see: Bernstein *et al.* (1995 ▶). For ring puckering parameters, see: Cremer & Pople (1975 ▶).
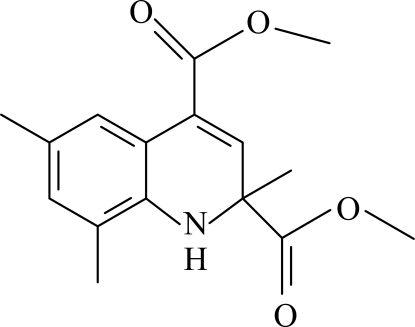


## Experimental

### 

#### Crystal data

C_16_H_19_NO_4_
*M* *_r_* = 289.33
Monoclinic, 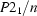

*a* = 7.7944 (5) Å
*b* = 23.4621 (8) Å
*c* = 8.2551 (5) Å
β = 93.729 (5)°
*V* = 1506.44 (14) Å^3^
*Z* = 4
Mo *K*α radiation
μ = 0.09 mm^−1^
*T* = 294 K
0.45 × 0.35 × 0.05 mm

#### Data collection

Nicolet P3 diffractometer
3188 measured reflections
2971 independent reflections
1999 reflections with *I* > 2σ(*I*)
*R*_int_ = 0.030
3 standard reflections every 50 reflections  intensity decay: 2%

#### Refinement

*R*[*F* ^2^ > 2σ(*F* ^2^)] = 0.056
*wR*(*F* ^2^) = 0.144
*S* = 1.07
2971 reflections
244 parameters
H atoms treated by a mixture of independent and constrained refinement
Δρ_max_ = 0.19 e Å^−3^
Δρ_min_ = −0.21 e Å^−3^

### 

Data collection: *XSCANS* (Siemens, 1996 ▶); cell refinement: *XSCANS*; data reduction: *SHELXTL* (Sheldrick, 2008 ▶); program(s) used to solve structure: *SHELXS97* (Sheldrick, 2008 ▶); program(s) used to refine structure: *SHELXL97* (Sheldrick, 2008 ▶); molecular graphics: *ORTEP-3 for Windows* (Farrugia, 1997 ▶) and *Mercury* (Macrae *et al.*, 2006 ▶); software used to prepare material for publication: *WinGX* publication routines (Farrugia, 1999 ▶).

## Supplementary Material

Crystal structure: contains datablocks I, global. DOI: 10.1107/S160053681004153X/su2218sup1.cif

Structure factors: contains datablocks I. DOI: 10.1107/S160053681004153X/su2218Isup2.hkl

Additional supplementary materials:  crystallographic information; 3D view; checkCIF report

## Figures and Tables

**Table 1:** Hydrogen-bond geometry (Å, °)

*D*—H⋯*A*	*D*—H	H⋯*A*	*D*⋯*A*	*D*—H⋯*A*
N1—H1⋯O1^i^	0.91 (3)	2.30 (3)	3.190 (3)	166 (3)
C2—H2⋯O4^ii^	0.94 (3)	2.54 (3)	3.444 (3)	162 (2)

Symmetry codes: (i) 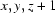; (ii) 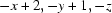.

**Fig. 1.**
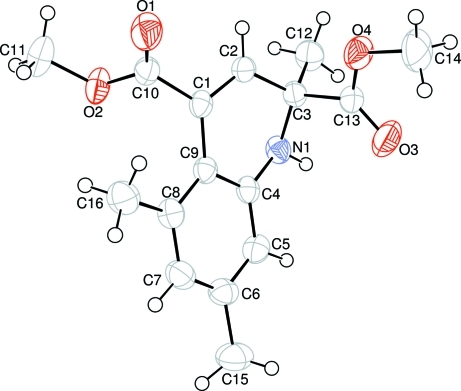
The molecular structure of the title molecule with the atom-numbering scheme. Displacement ellipsoids are drawn at the 50% probability level.

**Fig. 2.**
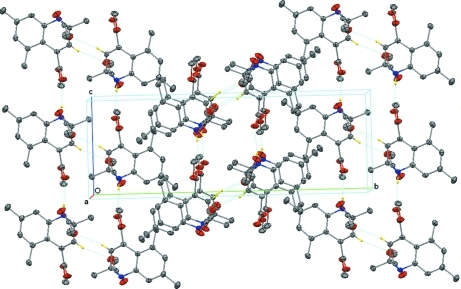
A partial packing diagram, viewed down the a-axis, of the title compound. Hydrogen bonds are shown as dashed cyan lines (see Table 1 for details).

## supplementary crystallographic information

### Comment

Dihydroquinolines have been widely studied and found an important structural unit in synthetic organic and medicinal chemistry (Elmore *et al.*, 2001; Dillard *et al.*, 1973; Muren & Weissmann, 1971). Many dihydroquinoline derivatives have been reported in the literature (Edwards *et al.*, 1998; Yan *et al.*, 2004; Petasis & Butkevich, 2009) and some of them have biological effects. For example, 2,2,4-substituted 1,2-dihydroquinolines have been shown antibacterial activities (Johnson *et al.*, 1989). They are also powerful intermediates for the preparation of quinolines (Dauphinee & Forrest, 1978; Yan *et al.*, 2004; Tom & Ruel, 2001; Tokuyama *et al.*, 2001) and 1,2,3,4-tetrahydroquinolines (Katritzky *et al.*, 1996). Many synthetic methods have been developed for the preparation of quinolines (Sarma & Prajapati, 2008; Martinez *et al.*, 2008; Huang *et al.*, 2009) and many quinolines display biological effects (Hamann *et al.*, 1998; He *et al.*, 2003; LaMontagne *et al.*, 1989).

In the title molecule, illustrated in Fig. 1, ring A (C1-C4/C9/N1) is not planar with the puckering parameters (Cremer & Pople, 1975) Q_T_ = 0.358 (2) Å, φ = 155.3 (4)° and θ = 67.1 (4)°.

In the crystal of the title compound intermolecular C-H···O hydrogen bonds (Table 1) link the molecules into R_2_^2^(10) dimers centered about an inversion center (Bernstein *et al.*, 1995). These dimers are further connected *via* intermolecular N-H···O hydrogen bonds (Table 1) to form infinite double chains propagating along [001] (Fig. 2).

### Experimental

The title compound was synthesized by the literature method (Waldmann *et al.*, 2008). 2,4-dimethylaniline (100 mg, 1 eq) was dissolved in chloroform (1.5 ml) in a screw-capped test tube and Bi(OTf)_3_ (5 mol%, 0.05 eq) was added to the mixture. The mixture was stirred at room temperature for 6 d until the starting material was completely consumed as monitored by TLC. The resultant residue was directly purified by flash chromatography on silica (EtOAc:Cylohexane 2:98) and gave, in 60% yield, a pale yellow solid. This solid was recrystallized over pentane and ethyl acetate (70:30) to give a pale yellow crystalline solid; R_f_ 0.25 (2:1 Cyclohexanone/EtOAc); mp. 420-421 K (Rueping & Gültekin, 2009).

### Refinement

The C14 and C16 methyl H-atoms were positioned geometrically and constrained to ride on their parent atoms: C-H = 0.96 Å with *U*_iso_(H) = *1.5U*_eq_(C). The remaining H-atoms were located in a difference Fourier map and were refined isotropically.

### Crystal data

**Table:**

C_16_H_19_NO_4_	*F*(000) = 616
*M**_r_* = 289.33	*D*_x_ = 1.276 Mg m^−^^3^
Monoclinic, *P*2_1_/*n*	Mo *K*α radiation, λ = 0.71073 Å
Hall symbol: -P 2yn	Cell parameters from 26 reflections
*a* = 7.7944 (5) Å	θ = 12–14°
*b* = 23.4621 (8) Å	µ = 0.09 mm^−^^1^
*c* = 8.2551 (5) Å	*T* = 294 K
β = 93.729 (5)°	Plate, pale yellow
*V* = 1506.44 (14) Å^3^	0.45 × 0.35 × 0.05 mm
*Z* = 4	

### Data collection

**Table:**

Nicolet P3 diffractometer	*R*_int_ = 0.030
Radiation source: fine-focus sealed tube	θ_max_ = 26.0°, θ_min_ = 1.7°
graphite	*h* = 0→9
Wyckoff–Scan scans	*k* = 0→28
3188 measured reflections	*l* = −10→10
2971 independent reflections	3 standard reflections every 50 reflections
1999 reflections with *I* > 2σ(*I*)	intensity decay: 2%

### Refinement

**Table:**

Refinement on *F*^2^	Primary atom site location: structure-invariant direct methods
Least-squares matrix: full	Secondary atom site location: difference Fourier map
*R*[*F*^2^ > 2σ(*F*^2^)] = 0.056	Hydrogen site location: inferred from neighbouring sites
*wR*(*F*^2^) = 0.144	H atoms treated by a mixture of independent and constrained refinement
*S* = 1.07	*w* = 1/[σ^2^(*F*_o_^2^) + (0.0504*P*)^2^ + 0.6624*P*] where *P* = (*F*_o_^2^ + 2*F*_c_^2^)/3
2971 reflections	(Δ/σ)_max_ < 0.001
244 parameters	Δρ_max_ = 0.19 e Å^−^^3^
0 restraints	Δρ_min_ = −0.21 e Å^−^^3^

### Special details

**Table:**

Geometry. All esds (except the esd in the dihedral angle between two l.s. planes) are estimated using the full covariance matrix. The cell esds are taken into account individually in the estimation of esds in distances, angles and torsion angles; correlations between esds in cell parameters are only used when they are defined by crystal symmetry. An approximate (isotropic) treatment of cell esds is used for estimating esds involving l.s. planes.
Refinement. Refinement of F^2^ against ALL reflections. The weighted R-factor wR and goodness of fit S are based on F^2^, conventional R-factors R are based on F, with F set to zero for negative F^2^. The threshold expression of F^2^ > 2sigma(F^2^) is used only for calculating R-factors(gt) etc. and is not relevant to the choice of reflections for refinement. R-factors based on F^2^ are statistically about twice as large as those based on F, and R- factors based on ALL data will be even larger.

### Fractional atomic coordinates and isotropic or equivalent isotropic displacement parameters (Å^2^)

**Table:**

	*x*	*y*	*z*	*U*_iso_*/*U*_eq_	
O1	0.7764 (3)	0.59692 (11)	−0.3063 (2)	0.0640 (6)	
O2	0.5173 (2)	0.61471 (9)	−0.2180 (2)	0.0502 (5)	
O3	1.1234 (3)	0.60687 (11)	0.3726 (3)	0.0738 (7)	
O4	1.1641 (2)	0.56175 (9)	0.1411 (2)	0.0539 (6)	
N1	0.7690 (3)	0.60501 (9)	0.3076 (3)	0.0393 (5)	
H1	0.792 (4)	0.6030 (12)	0.417 (4)	0.054 (9)*	
C1	0.7476 (3)	0.61554 (11)	−0.0238 (3)	0.0334 (5)	
C2	0.8247 (3)	0.56972 (11)	0.0436 (3)	0.0352 (6)	
H2	0.849 (3)	0.5376 (11)	−0.018 (3)	0.035 (7)*	
C3	0.8769 (3)	0.56643 (10)	0.2214 (3)	0.0353 (6)	
C4	0.7347 (3)	0.65906 (11)	0.2432 (3)	0.0341 (5)	
C5	0.7101 (3)	0.70451 (12)	0.3481 (3)	0.0415 (6)	
H5	0.733 (3)	0.6993 (10)	0.464 (3)	0.033 (6)*	
C6	0.6669 (3)	0.75784 (12)	0.2865 (3)	0.0447 (7)	
C7	0.6494 (4)	0.76513 (12)	0.1202 (4)	0.0466 (7)	
H7	0.620 (3)	0.8011 (12)	0.074 (3)	0.046 (8)*	
C8	0.6763 (3)	0.72106 (11)	0.0118 (3)	0.0403 (6)	
C9	0.7161 (3)	0.66663 (11)	0.0741 (3)	0.0342 (5)	
C10	0.6878 (3)	0.60882 (11)	−0.1978 (3)	0.0389 (6)	
C11	0.4423 (5)	0.60908 (19)	−0.3831 (4)	0.0605 (9)	
H111	0.475 (5)	0.6429 (16)	−0.448 (4)	0.084 (12)*	
H112	0.485 (5)	0.5743 (16)	−0.428 (4)	0.077 (12)*	
H113	0.322 (6)	0.6056 (16)	−0.371 (5)	0.095 (13)*	
C12	0.8522 (4)	0.50594 (13)	0.2861 (4)	0.0500 (7)	
H121	0.873 (4)	0.5072 (12)	0.407 (4)	0.057 (9)*	
H123	0.734 (4)	0.4934 (13)	0.252 (4)	0.064 (9)*	
H122	0.926 (4)	0.4786 (14)	0.238 (4)	0.070 (10)*	
C13	1.0681 (3)	0.58165 (11)	0.2556 (3)	0.0376 (6)	
C14	1.3481 (4)	0.57041 (17)	0.1667 (5)	0.0720 (10)	
H14A	1.4029	0.5604	0.0698	0.108*	
H14B	1.3924	0.5468	0.2549	0.108*	
H14C	1.3709	0.6097	0.1924	0.108*	
C15	0.6472 (6)	0.80746 (16)	0.4010 (5)	0.0637 (9)	
H151	0.582 (6)	0.7969 (18)	0.496 (5)	0.108 (15)*	
H152	0.588 (6)	0.840 (2)	0.347 (5)	0.115 (16)*	
H153	0.754 (6)	0.8168 (18)	0.450 (5)	0.109 (16)*	
C16	0.6685 (4)	0.73525 (12)	−0.1667 (3)	0.0536 (8)	
H16A	0.7586	0.7154	−0.2172	0.080*	
H16B	0.6828	0.7756	−0.1802	0.080*	
H16C	0.5591	0.7238	−0.2163	0.080*	

### Atomic displacement parameters (Å^2^)

**Table:**

	*U*^11^	*U*^22^	*U*^33^	*U*^12^	*U*^13^	*U*^23^
O1	0.0579 (13)	0.0996 (18)	0.0346 (11)	0.0201 (12)	0.0036 (9)	−0.0042 (11)
O2	0.0363 (10)	0.0755 (14)	0.0376 (10)	−0.0025 (9)	−0.0070 (8)	0.0008 (9)
O3	0.0470 (12)	0.106 (2)	0.0664 (15)	−0.0009 (12)	−0.0122 (11)	−0.0370 (14)
O4	0.0303 (9)	0.0749 (14)	0.0564 (12)	−0.0054 (9)	0.0028 (8)	−0.0166 (10)
N1	0.0414 (12)	0.0463 (13)	0.0301 (11)	0.0068 (10)	0.0026 (9)	0.0048 (10)
C1	0.0252 (11)	0.0435 (14)	0.0316 (12)	−0.0012 (10)	0.0020 (9)	−0.0001 (10)
C2	0.0292 (12)	0.0376 (14)	0.0385 (14)	−0.0003 (10)	−0.0001 (10)	−0.0036 (11)
C3	0.0321 (13)	0.0362 (13)	0.0372 (13)	0.0009 (10)	−0.0006 (10)	0.0014 (11)
C4	0.0245 (11)	0.0414 (14)	0.0365 (12)	0.0002 (10)	0.0029 (9)	0.0015 (11)
C5	0.0391 (14)	0.0533 (17)	0.0326 (14)	0.0000 (12)	0.0064 (11)	−0.0036 (12)
C6	0.0367 (14)	0.0442 (16)	0.0538 (17)	0.0013 (12)	0.0085 (12)	−0.0081 (13)
C7	0.0454 (15)	0.0377 (15)	0.0563 (18)	0.0041 (12)	0.0013 (13)	0.0031 (13)
C8	0.0360 (13)	0.0437 (15)	0.0411 (14)	0.0009 (11)	0.0015 (11)	0.0032 (12)
C9	0.0282 (11)	0.0398 (14)	0.0344 (13)	0.0003 (10)	0.0016 (10)	−0.0004 (10)
C10	0.0385 (13)	0.0449 (15)	0.0330 (13)	0.0026 (11)	−0.0014 (11)	−0.0008 (11)
C11	0.056 (2)	0.078 (3)	0.0452 (18)	−0.0146 (19)	−0.0160 (15)	0.0065 (18)
C12	0.0518 (18)	0.0420 (17)	0.0555 (19)	−0.0044 (14)	−0.0012 (15)	0.0088 (14)
C13	0.0371 (13)	0.0382 (14)	0.0365 (13)	0.0019 (11)	−0.0047 (10)	0.0003 (11)
C14	0.0314 (15)	0.102 (3)	0.083 (2)	−0.0042 (17)	0.0006 (15)	−0.008 (2)
C15	0.068 (2)	0.055 (2)	0.070 (2)	0.0065 (18)	0.014 (2)	−0.0184 (18)
C16	0.066 (2)	0.0452 (17)	0.0492 (17)	0.0033 (14)	0.0014 (14)	0.0094 (13)

### Geometric parameters (Å, °)

**Table:**

O1—C10	1.199 (3)	C6—C15	1.513 (4)
O2—C10	1.336 (3)	C7—H7	0.95 (3)
O2—C11	1.454 (3)	C8—C7	1.392 (4)
O3—C13	1.189 (3)	C8—C16	1.508 (4)
O4—C13	1.328 (3)	C9—C8	1.404 (3)
O4—C14	1.450 (3)	C11—H111	1.00 (4)
N1—C3	1.453 (3)	C11—H112	0.96 (4)
N1—C4	1.394 (3)	C11—H113	0.95 (4)
N1—H1	0.91 (3)	C12—H121	1.01 (3)
C1—C2	1.335 (3)	C12—H122	0.97 (3)
C1—C9	1.475 (3)	C12—H123	0.99 (3)
C1—C10	1.490 (3)	C14—H14A	0.9600
C2—H2	0.94 (3)	C14—H14B	0.9600
C3—C2	1.499 (3)	C14—H14C	0.9600
C3—C12	1.533 (4)	C15—H151	0.99 (4)
C3—C13	1.541 (3)	C15—H153	0.93 (5)
C4—C5	1.395 (4)	C15—H152	0.98 (5)
C4—C9	1.405 (3)	C16—H16A	0.9600
C5—H5	0.97 (2)	C16—H16B	0.9600
C6—C5	1.384 (4)	C16—H16C	0.9600
C6—C7	1.381 (4)		
			
C10—O2—C11	116.3 (2)	O1—C10—C1	125.8 (2)
C13—O4—C14	116.4 (2)	O2—C10—C1	110.9 (2)
C3—N1—H1	111.9 (19)	O2—C11—H111	109 (2)
C4—N1—C3	118.9 (2)	O2—C11—H112	108 (2)
C4—N1—H1	116.5 (18)	O2—C11—H113	104 (2)
C2—C1—C9	120.9 (2)	H111—C11—H112	111 (3)
C2—C1—C10	114.9 (2)	H113—C11—H111	114 (3)
C9—C1—C10	124.1 (2)	H113—C11—H112	110 (3)
C1—C2—C3	122.4 (2)	C3—C12—H121	107.6 (17)
C1—C2—H2	121.5 (15)	C3—C12—H122	112.1 (19)
C3—C2—H2	116.2 (15)	C3—C12—H123	108.2 (18)
N1—C3—C2	108.6 (2)	H121—C12—H122	112 (3)
N1—C3—C12	108.4 (2)	H121—C12—H123	112 (2)
N1—C3—C13	110.5 (2)	H123—C12—H122	105 (3)
C2—C3—C12	110.9 (2)	O3—C13—O4	124.2 (2)
C2—C3—C13	111.4 (2)	O3—C13—C3	124.0 (2)
C12—C3—C13	107.0 (2)	O4—C13—C3	111.8 (2)
N1—C4—C5	119.3 (2)	O4—C14—H14A	109.5
N1—C4—C9	119.9 (2)	O4—C14—H14B	109.5
C5—C4—C9	120.7 (2)	O4—C14—H14C	109.5
C4—C5—H5	119.5 (15)	H14A—C14—H14B	109.5
C6—C5—C4	120.2 (2)	H14A—C14—H14C	109.5
C6—C5—H5	120.1 (15)	H14B—C14—H14C	109.5
C5—C6—C15	119.9 (3)	C6—C15—H151	112 (3)
C7—C6—C5	118.9 (3)	C6—C15—H152	112 (3)
C7—C6—C15	121.2 (3)	C6—C15—H153	109 (3)
C6—C7—C8	122.5 (3)	H151—C15—H152	108 (4)
C6—C7—H7	121.0 (16)	H151—C15—H153	102 (4)
C8—C7—H7	116.5 (16)	H153—C15—H152	113 (4)
C7—C8—C9	118.6 (2)	C8—C16—H16A	109.5
C7—C8—C16	117.8 (2)	C8—C16—H16B	109.5
C9—C8—C16	123.5 (2)	C8—C16—H16C	109.5
C4—C9—C1	115.5 (2)	H16A—C16—H16B	109.5
C8—C9—C1	125.4 (2)	H16A—C16—H16C	109.5
C8—C9—C4	119.0 (2)	H16B—C16—H16C	109.5
O1—C10—O2	123.2 (2)		
			
C11—O2—C10—O1	−3.4 (4)	N1—C3—C13—O3	23.5 (4)
C11—O2—C10—C1	180.0 (3)	N1—C3—C13—O4	−158.4 (2)
C14—O4—C13—O3	2.1 (4)	C2—C3—C13—O3	144.2 (3)
C14—O4—C13—C3	−176.1 (2)	C2—C3—C13—O4	−37.6 (3)
C4—N1—C3—C2	−43.0 (3)	C12—C3—C13—O3	−94.4 (3)
C4—N1—C3—C12	−163.6 (2)	C12—C3—C13—O4	83.7 (3)
C4—N1—C3—C13	79.5 (3)	N1—C4—C5—C6	−176.6 (2)
C3—N1—C4—C5	−148.5 (2)	C9—C4—C5—C6	0.0 (4)
C3—N1—C4—C9	34.8 (3)	N1—C4—C9—C1	−4.2 (3)
C9—C1—C2—C3	2.5 (4)	N1—C4—C9—C8	178.1 (2)
C10—C1—C2—C3	−173.5 (2)	C5—C4—C9—C1	179.2 (2)
C2—C1—C9—C4	−14.1 (3)	C5—C4—C9—C8	1.5 (4)
C2—C1—C9—C8	163.4 (2)	C7—C6—C5—C4	−0.3 (4)
C10—C1—C9—C4	161.5 (2)	C15—C6—C5—C4	−177.6 (3)
C10—C1—C9—C8	−21.0 (4)	C5—C6—C7—C8	−1.0 (4)
C2—C1—C10—O1	−58.1 (4)	C15—C6—C7—C8	176.3 (3)
C2—C1—C10—O2	118.4 (2)	C9—C8—C7—C6	2.6 (4)
C9—C1—C10—O1	126.0 (3)	C16—C8—C7—C6	−174.9 (3)
C9—C1—C10—O2	−57.5 (3)	C1—C9—C8—C7	179.8 (2)
N1—C3—C2—C1	24.7 (3)	C1—C9—C8—C16	−2.9 (4)
C12—C3—C2—C1	143.8 (3)	C4—C9—C8—C7	−2.7 (4)
C13—C3—C2—C1	−97.2 (3)	C4—C9—C8—C16	174.5 (2)

### Hydrogen-bond geometry (Å, °)

**Table:**

*D*—H···*A*	*D*—H	H···*A*	*D*···*A*	*D*—H···*A*
N1—H1···O1^i^	0.91 (3)	2.30 (3)	3.190 (3)	166 (3)
C2—H2···O4^ii^	0.94 (3)	2.54 (3)	3.444 (3)	162 (2)

Symmetry codes: (i) *x*, *y*, *z*+1; (ii) −*x*+2, −*y*+1, −*z*.
